# Molecular Targets for PET Imaging of Activated Microglia: The Current Situation and Future Expectations

**DOI:** 10.3390/ijms18040802

**Published:** 2017-04-11

**Authors:** Claire Tronel, Bérenger Largeau, Maria Joao Santiago Ribeiro, Denis Guilloteau, Anne-Claire Dupont, Nicolas Arlicot

**Affiliations:** 1 INSERM U930, Université François Rabelais de Tours, 10 boulevard Tonnelé, 37032 Tours, France; maria.ribeiro@univ-tours.fr (M.J.S.R.); denis.guilloteau@univ-tours.fr (D.G.); ac.dupont@chu-tours.fr (A.-C.D.); nicolas.arlicot@univ-tours.fr (N.A.); 2 CHRU de Tours, 37044 Tours, France; berenger.largeau@etu.univ-tours.fr

**Keywords:** microglial activation, neuroinflammation, PET, biomarker, neurodegenerative disorders

## Abstract

Microglia, as cellular mediators of neuroinflammation, are implicated in the pathogenesis of a wide range of neurodegenerative diseases. Positron emission tomography (PET) imaging of microglia has matured over the last 20 years, through the development of radiopharmaceuticals targeting several molecular biomarkers of microglial activation and, among these, mainly the translocator protein-18 kDa (TSPO). Nevertheless, current limitations of TSPO as a PET microglial biomarker exist, such as low brain density, even in a neurodegenerative setting, expression by other cells than the microglia (astrocytes, peripheral macrophages in the case of blood brain barrier breakdown), genetic polymorphism, inducing a variation for most of TSPO PET radiopharmaceuticals’ binding affinity, or similar expression in activated microglia regardless of its polarization (pro- or anti-inflammatory state), and these limitations narrow its potential interest. We overview alternative molecular targets, for which dedicated radiopharmaceuticals have been proposed, including receptors (purinergic receptors P2X7, cannabinoid receptors, α7 and α4β2 nicotinic acetylcholine receptors, adenosine 2A receptor, folate receptor β) and enzymes (cyclooxygenase, nitric oxide synthase, matrix metalloproteinase, β-glucuronidase, and enzymes of the kynurenine pathway), with a particular focus on their respective contribution for the understanding of microglial involvement in neurodegenerative diseases. We discuss opportunities for these potential molecular targets for PET imaging regarding their selectivity for microglia expression and polarization, in relation to the mechanisms by which microglia actively participate in both toxic and neuroprotective actions in brain diseases, and then take into account current clinicians’ expectations.

## 1. Microglia: From Resting to Activated Phenotypes

Microglia cells were first identified by Nissl in 1899, who already suggested that they can exert phagocytosis and migration [1]. Pio del Rio-Hortega, in 1921, defined them as cells with ramifications, able to transform after a pathologic event and to acquire ameboid morphology, as well as to migrate, proliferate, and exert phagocytosis [2]. Microglial cells are resident immune cells of the brain and the most important effector of brain innate immunity. Their capacities (motility, proliferation, phagocytosis, secretion of soluble molecules, etc.) are very close to those of macrophages, and microglial cells are often called “brain macrophages”. Nevertheless, after 50 years of debate, it is now commonly recognized that microglia and macrophages have different genesis. Macrophages are produced in the bone marrow from the post-natal stage, whereas microglia are derived from yolk sac progenitors migrating in the neuroepithelium at the early stage of embryonic life [3,4]. Despite this difference, these two types of cells require the same proliferation and differentiation factors and share identical receptors (CD11b, CD14, etc.).

Microglial cells represent between 5% and 10% of adult brain cells. They are present in all brain areas, but with variable density, even in physiological conditions, up to 10-fold in the human brain [5]. Anatomically, microglia cells are more expressed in the telencephalon or diencephalon than mesencephalon [6]. Their repartition varies also between gray and white matter and according to axons’ myelination, since myelinated parts of the brain have a higher density of microglial cells than non-myelinated areas in the same brain region [7]. A recent study in rhesus macaques points out that microglial cell density, especially in gray matter, is modified during the life-span and increases with aging [8]. Furthermore, as reported by Pintado and colleagues [9], in rats, following brain lipopolysaccharide (LPS) injection, differences in microglia brain distribution, density, and functionality were associated with different sites of injection. More recently, a genome-wide study on mice showed that microglia from several brain regions and at different ages presented differences of gene expression [10].

Kreutzberg [11] was the first to characterize microglia morphological changes occurring during microglia activation. In mature and healthy central nervous systems (CNS), microglia present a ramified morphology, with a small soma and thin cellular processes. This state corresponds to “quiescent” or “resting” microglia. Resting microglia express few surface markers and have been considered for years as functionally inactive. Nonetheless, in vivo study on microglia-GFP (green fluorescent protein) transgenic mice showed that these ramifications have a high motility allowing microglial cells to survey their environment in an active way [12]. Microglial cells are sensors of brain integrity, since any change of brain homeostasis will induce their activation through a modification of gene expression, morphology, and function [13,14]. Activated microglia show several morphological changes. First, the cell ramification number is increased and these ramifications are thicker, so that cells acquire an amoeboid phenotype, very close to peripheral macrophages [15].

For years, microglia activation was supposed to be a “yes or no” phenomenon, with cells only known to be resting or activated. In the last ten years, it has become clear that microglia activation processes are much more complex. As well as peripheral macrophages, microglial cell activation is a sequential process, leading to distinct phenotypes and functions of these cells depending on the stimulus leading to their activation [16,17]. The classification and characterization of the different subpopulation of microglial cells is based on these definitions for peripheral macrophages [18]. Classically or pro-inflammatory (M1) activated microglia are activated by LPS and interferon γ (IFNγ). M1 microglial cells secrete reactive oxygen species (ROS), as well as pro-inflammatory cytokines, such as interleukin (IL)-1β and tumor necrosis factor (TNF)-α. Their activation may lead to tissue inflammation [19,20]. Alternative or anti-inflammatory microglial cells (M2) are divided into three subpopulations. M2a cells are activated by IL-4 or IL-13 and produce growth factors (insulin growth factor (IGF)-1) and anti-inflammatory cytokines (IL-10). They participate in tissue reparation, and also have an important phagocytosis capacity, involving them in the cleaning of cellular debris [21,22]. M2b cells are induced by immune complexes, secrete IL-10, and regulate the immune response [23]. Finally, IL-10, or glucocorticoids, will lead to the M2c phenotype, also called “acquired deactivation”, producing tumor growth factor-(TGF)β [24]. As brain immune cells, the principal function of microglia is to protect the brain against injury. They are the first actors in brain inflammation, even if other cells, mostly astrocytes, are also involved.

## 2. Involvement of Activated Microglia in Brain Disorders

Microglia activation is basically a beneficial phenomenon in response to neuron injury. Microglial cells phagocytose apoptotic neurons and secrete inflammatory factors to attract other immune cells to the site of injury. Inflammation resolution is well regulated and occurs when injury has been treated [25]. Nevertheless, it is now clearly established that neuroinflammation takes part in the process of most brain diseases, either in acute (traumatic brain injury (TBI), stroke) or in chronic, neurodegenerative diseases. Glutamate, adenosine triphosphate (ATP), chemokines, or superoxide are released by apoptotic brain cells (neurons, astrocytes) and lead to microglia activation through specific receptors.

In stroke or TBI, microglia activation appears in the hours following injury, release pro-inflammatory factors and chemoattractant molecules recruiting peripheral immune cells (macrophages and lymphocytes), and participate to neuronal death. Moreover, chronic neuronal death takes place in the weeks or months after these injuries and is strongly associated with microglia activation [26,27]. Molecules with anti-inflammatory effects have shown neuroprotection in preclinical models of stroke [28] and TBI [29]. These preclinical results lead to clinical study using anti-inflammatory molecules on stroke patients. Results of these clinical trials are variable and reviewed in Veltkamp and Gill [30].

In neurodegenerative diseases, the first involvement of neuroinflammation was described in Alzheimer’s disease (AD) patients for whom activated microglial cells were detected in post-mortem nearby senile plaques [26]. Later, epidemiological studies suggest that long-term daily consumption of non-steroidal anti-inflammatory drugs (NSAIDs) reduce the risks of AD and Parkinson’s (PD) diseases’ development [27,31,32,33]. Since then, neuroinflammation and microglia are a rich topic of research for neurodegenerative disease treatment and diagnosis. In these diseases, protein aggregates, such as Aβ-amyloid deposits in AD or α-synuclein in PD, will also activate microglia [34,35]. The persistence of these signals leads, in the long term, to an uncontrolled and unregulated microglia activation with high secretion of inflammatory factors which, in turn, will actively participate to neuronal death, because these cells are particularly sensitive to oxidative stress [36].

For years, targeting microglia in neurodegenerative diseases consisted in attempts to decrease cell activation and interesting results were obtained in preclinical models of AD, PD, and amyotrophic lateral sclerosis (ALS) [37,38,39,40,41]. However, no clinical effects of anti-inflammatory treatment were observed in clinical trials [42,43,44]. According to the recent knowledge on microglia polarization, promoting M2-microglia phenotype may be a promising target, as shown by studies on preclinical models of stroke [45], TBI [46], AD [47], PD [48], ALS [49], and multiple sclerosis (MS) [50]. Nonetheless, targeting neuroinflammation in these diseases is even more complex and a regimen of anti-inflammatory treatments is also crucial. In fact, at the early stage of neurodegenerative diseases, microglia activation might exert a neuroprotective effect [51,52].

## 3. Imaging of Activated Microglia

Regarding this activated microglia involvement in neurodegenerative disorders, and its potential therapeutic impact [31,32,33], interest in the development of suitable imaging tools to investigate microglia as a relevant marker of neuronal damage and CNS activity [53] has risen in recent years. Thus, in vivo imaging of activated microglia can provide a non-invasive and reliable detection of early and localized neuroinflammation processes, thanks to the availability of several neuroimaging modalities. On one hand, MRI-based (magnetic resonance imaging) techniques for the detection of neuroinflammation have been developed, based on the macrophages’ labelling by ultrasmall superparamagnetic iron oxide (USPIO) nanoparticles [54]. Targeting neuroinflammatory changes with USPIO has been used in the clinical setting, where no correlation was found between iron oxide-based enhancement and infarct size in human studies [55]. This approach reflects vascular integrity rather than microglial activation, and the specificity and accuracy of labelling strategies in molecular MRI remain to be validated. On the other hand, in vivo functional cerebral imaging of activated microglia has been widely explored by positron emission tomography (PET) molecular imaging, using sensitive radioactive probes targeting specific molecular mediators of the inflammation cascade (cell surface and mitochondrial receptors or transporters expressed in activated microglia). We will outline an overview of these molecular targets, focusing on their respective contribution for the understanding of microglial involvement in neurodegenerative diseases.

## 4. 18-kDa Translocator Protein (TSPO): The “Gold Standard” Molecular Target for Activated Microglia PET Imaging

### 4.1. TSPO

The 18-kDa translocator protein (TSPO) is a hetero-oligomeric complex located on the outer mitochondrial membrane known to be involved in modulating immune response, in cholesterol transport, and in heme/steroid synthesis [56]. TSPO was first described as a peripheral benzodiazepine receptor (PBR), a secondary binding site for diazepam, but has been renamed to TSPO to reflect some of these cellular functions [57]. This protein was initially found in peripheral organs (i.e., kidneys, nasal epithelium, adrenal glands, lungs, and heart), but is also minimally expressed on microglia in healthy brains [58]. However, TSPO expression is dramatically upregulated during the microglia activation process. Its basal expression rises in several acute and degenerative disorders, including AD, PD, MS, Huntington’s disease (HD) [59], and amyotrophic lateral sclerosis (ALS) [60]. As a result, TSPO has been considered a hallmark of neuroinflammation. Therefore, TSPO PET imaging has been used for both improving the knowledge regarding the role of neuroinflammation in CNS diseases and to assess the efficacy of novel anti-inflammatory therapeutic strategies. The most widely used TSPO PET radiopharmaceutical, namely ^11^C-(R)-PK11195, is an isoquinoline carboxamide developed in the early 1980s [61]. However, ^11^C-(R)-PK11195’s clinical usefulness is narrowed by several major limitations, including the short half-life of carbon-11, a low brain bioavailability and a poor signal-to-noise ratio due to high nonspecific binding [62]. To counteract these drawbacks, there has been a great amount of effort toward the development of second-generation TSPO PET radiotracers [63,64,65], including ^18^F-FEDAA1106, ^11^C-PBR28, ^11^C-DPA-713, and ^18^F-DPA-714.

TSPO PET radioligands made it possible to characterize in vivo, in numerous preclinical and clinical studies, the neuroinflammatory component of neurodegenerative disorders its spatial distribution, its intensity, and its longitudinal evolution. Thus, TSPO PET imaging is, nowadays, widely recognized as a useful biomarker of activated microglia involvement in CNS disorders that assisted in the early detection of neuroinflammation, monitor the severity and progression of the neurodegenerative diseases, and help to consider the effectiveness of emerging CNS therapies aimed at decreasing neuroinflammation.

### 4.2. TSPO Limitations as a Molecular Target for Activated Microglia PET Imaging

Several drawbacks limit the ability of TSPO as PET imaging molecular target.

First, a genetic polymorphism in exon 4 of the TSPO gene (*rs6971*) has been identified, resulting in an alanine-to-threonine substitution (A147T) [66,67]. This polymorphism affects the binding affinity properties of most of PET TSPO radiopharmaceuticals for their target. Sensitivity to TSPO polymorphism is variable depending on the tracer considered , resulting in a very large heterogeneity in PET images and their associated quantitative data [66]. Three distinct binder statuses have been identified: HAB, high- (A/A; ~70%), MAB, mixed- (A/T; ~21%), and LAB, low-affinity binders (T/T; ~9%). Then, this polymorphism, and its consequent binder status, can be identified by genetic analysis allowing stratification of subjects, and can subsequently account for binder status in the quantification of TSPO PET studies using second-generation radiotracers [68]. However, in LAB patients, TSPO PET images are of significantly lower quality, and the clinical usefulness of this approach is, therefore, limited in these patients. Even if the conclusions of clinical studies performed only on HAB or MAB patients might be extended to a whole-patient population, at least in AD [69], this polymorphism makes more difficult the design of clinical trials based on TSPO PET imaging. A third-generation TSPO radioligand, namely ^11^C-ER176, sensitive to polymorphism in vivo but allowing quantification in LAB patients, has recently been proposed; nonetheless, the clinical relevance of this compound remains to be confirmed [70].

Secondly, the mathematical model usually applied to quantify a brain PET radioligand binding requires either serial blood samples or a reference region free of specific ligand binding. However, TSPO is distributed throughout the entire brain, even at very low density, and no clear reference region may exist in neurodegenerative diseases. However, the cerebellum was validated as a reference region for ^11^C-PBR28-TSPO binding in AD patients [71], but regarding differences between radiotracers’ pharmacokinetics and the variety of TSPO distribution depending on the considered disease, it is necessary to independently validate a reference region for each radiotracer and for each disease.

A third concern for the meaning of this PET imaging approach is the multicellular expression of TSPO in the human brain. In addition to activated microglia, astroglial expression of TSPO, and, in the case of a disrupted blood brain barrier (BBB), on infiltrating cells of mononuclear-phagocyte lineage have been reported [72]. TSPO in vivo PET imaging does not strictly reflect the activation of microglial cells, but a broader inflammatory process [73]. The PET signal can also be disrupted by TSPO peripheral vascular endothelial cells’ expression, which, together with TSPO radioligands’ plasma protein binding, impacts a partial volume effect, especially for cortical areas close to large blood vessels. Thus, Rizzo et al. have studied the positive impact of considering a model that includes an additional irreversible compartment from the blood to the endothelium (vascular component) on the quantification of ^11^C-PBR28 data, compared to the standard two-tissue compartmental model (2TCM) [74]. Authors demonstrated that the inclusion of the vascular component in the kinetic model (2TCM-1K) provided a more precise and accurate quantification of ^11^C-PBR28 brain PET data. The estimates are more than three-fold smaller, have a higher time stability and are better correlated to brain mRNA TSPO expression with 2TCM-1K model compared to 2TCM [74].

Lastly, since the characterization of the different subpopulations of microglial cells toward different phenotypes (M1, neurotoxic vs. M2, neuroprotective, see Section 1), it has been challenging to identify and differentiate these subtypes and their related pro/anti-inflammatory roles for both physiopathological understanding and new therapeutic approach designs. While the advent of TSPO PET agents has enabled the distribution of activated microglia in the brain to be imaged in vivo, TSPO ligands bind to both M1 and M2 phenotypes. TSPO PET, therefore, provides a measure of activated microglia load without information related with its specific functional role in different diseases and brain areas [75,76].

## 5. Potential Alternative Molecular Targets for Activated Microglia Imaging

Limitations of the TSPO, as described above, have led to the identification of other molecular targets to develop new tracers of activated microglia. Moreover, in order to fulfill the current clinician’s expectancies regarding the microglial role in neurodegenerative diseases and associated potential novel therapeutic approaches, radiopharmaceuticals able to discriminate activated microglia according to their polarization from M1 to M2 phenotypes are, to date, highly expected. In this part, we summarize potential microglial imaging targets as follows: (1) Targets evaluated by PET in pre-clinical or clinical settings for imaging microglial cells within the CNS; (2) Targets that have been used for imaging inflammatory conditions by PET in peripheral disorders; and (3) Potential targets for which radioligands have not been tested/synthetized yet. We will also pay particular attention to the potential interest of these targets to discriminate microglia subtypes (see Table 1). However, no in vivo study using any PET radioligands allowed the study of only one subpopulation of microglia. Targeting these molecules for PET imaging of M1 or M2 microglia requires further exploration.

### 5.1. Molecular Targets Evaluated in CNS Diseases

Table 1 summarizes candidate microglial imaging targets, for which dedicated PET radiopharmaceuticals have been developed and evaluated in pre-clinical and/or clinical CNS settings.

#### 5.1.1. Cyclooxygenase (COX)

Cyclooxygenase is an enzyme producing important biological mediators, including prostaglandins, which are involved in the regulation of neuroinflammatory process in connection with neurodegenerative diseases [90,91,92].

Among the different COX isoforms characterized, COX-1 is classically described as a constitutively expressed house-keeping enzyme, whereas COX-2 is an inflammatory inducible isoform, mainly expressed in response to neuroinflammation [93]. COX-1 immunoreactivity is enriched in the midbrain, pons, and medulla [94], whereas COX-2 immunoreactivity prevails in neurons and glial cells of the hippocampus, hypothalamus, and amygdala [95,96]. COX-2 is, therefore, considered as a key player in the pathophysiology of AD, PD [97,98], and has been identified as a molecular target of interest for pharmacological design of selective ligands for both therapy and molecular imaging. Thus, highly-selective COX-2 radioligands, such as ^11^C-Celecoxib [99] or ^11^C-Rofecoxib [77], have been evaluated to explore microglial activation by PET, but to date this approach remains unsuccessful, due to either non-specific bindings or low in vivo sensitivity of these radioligands [100,101]. In this context, the development of a selective COX-1 imaging probe has regained interest. The ^11^C-ketoprofen methyl ester (^11^C-KTP-Me) has been evaluated in rodent models of focal neuroinflammation (intrastriatal injection of lipopolysaccharide or quinolinic acid), and exhibited striatal accumulation corresponding to the early phase of microglia activation (from six hours and at day 1 after the lesion) [102]. In contrast, the time course of striatal accumulation of ^11^C-PK11195, the gold-standard TSPO PET radioligand, started later and lasted up to 14 days afterward, corresponding to changes in activation of both microglia and astrocytes. This finding suggests the high specificity of cellular expression of COX-1 within microglia, during an acute neuroinflammatory process, and its ability to be evidenced by PET in vivo. More recently, the same group used ^11^C-KTP-Me to investigate COX-1 involvement in amyloid precursor protein transgenic (APPSWE2576) mice, an animal model of AD [78]. PET images of (*S*)-^11^C-KTP-Me specifically detected and clearly visualized the changes in COX-1 expression in activated microglia concomitantly to the formation of amyloid plaques in amyloid peptide precursor-transgenic mice (APP-Tg) mice. These preclinical data suggest that PET imaging of COX-1 with (*S*)-^11^C-KTP-Me could be a promising approach for monitoring activated microglia in CNS diseases, including AD.

#### 5.1.2. Cannabinoid Receptor

While the cannabinoid receptor type 1 (CB1R) is constitutively the most abundantly expressed G-protein-coupled receptor in the human brain [103], the inducible isoform, namely cannabinoid receptor type 2 (CB2R), is barely detectable in the healthy brain [104]. Low levels of CB2R expression have been found in microglial cells [104,105], in human fetal astrocytes [106], and in human cerebral microvascular endothelial cells [107]. Nonetheless, several studies reported an upregulation of CB2R on activated microglial cells in pathological conditions, including MS, ALS, PD, or AD [105,108,109,110]. Moreover, selective CB2R activation results in a decrease of microglial activation in HD and ALS transgenic mouse models and appears to be effective in reducing neurodegeneration [109,110]. Neuroprotective effects of CB2 agonists are associated with suppression of microglia activation via inhibiting the release of neurotoxic factors and by decreasing neuronal cell damage in cell or tissue culture models [111]. These observations suggest that therapeutic modulation of CB2R may be a new promising treatment for neuropathogenic disorders characterized by a neuroinflammatory component. Recent findings have indicated that nicotine attenuates Aβ-induced microglial activation by shifting microglial M1 to M2 state, and cannabinoid CB2R mediates the process, thereby suggesting the CB2R involved in microglia polarization shift [112]. Several CB2R selective ligands have been developed over the past years [113] and the preliminary clinical evaluation with ^11^C-NE40 showed appropriate fast brain kinetics in the healthy human brain [114]. However, ^11^C-NE40 has not succeeded in highlighting microglial activation in AD subjects as compared to healthy controls. In 2015, Slavik et al. reported a novel carbon-11 radiolabeled tracer ^11^C-*RS*-016 for CB2R imaging, which showed higher specific binding in postmortem ALS patient spinal cord tissues [79,80]. Since then, several other recently synthetized radiotracers are under preclinical investigations and seem to offer promising prospects for imaging CB2R expression [115,116,117].

#### 5.1.3. Purinergic Ion Channel Receptor

Purinergic ion channel receptor (P2X) is a large family of receptors distributed in a wide variety of tissue [118]. Among them, P2X7 receptor (P2X7R) are expressed both peripherally and in the CNS, especially in microglia, astrocytes and Schwann cells [119]. P2X7R activation is associated with production of pro-inflammatory cytokines (IL-1β) and ROS by peripheral macrophages, as well as microglia or astrocytes [120,121]. Moreover, P2X7R expression is increased in microglia in animal models of neurodegenerative diseases, such as AD [122], ALS [123], or HD [124]. Recently, P2X7R has been proposed as a marker of M1 microglia. Indeed, in a mouse ALS model, P2X7R inhibition led to a diminution of microgliosis associated with a decrease of M1 (IL-1β) and an increase of M2 (IL-10) markers, leading authors to consider P2X7R as a potential maker of M1 microglia in ALS [125]. Involvement of P2X7R in microglia M1 polarization has recently been confirmed in vitro as P2X7R inhibition avoid M1 microglia polarization in ischaemic conditions [126]. Nevertheless, in vitro, P2X7R expression is also reported in M2 polarized macrophages and might so play a role in inflammation resolution [127].

The first candidate was the P2X7R antagonist A-740003, showing a high affinity and selectivity for the receptor. Preclinical in vivo study of ^11^C-A-740003 showed little uptake in rat brains [81]. More recently, two other P2X7R antagonists has been radiolabeled and used in preclinical models. ^11^C-JNJ-54173717 was shown to cross the BBB in rats and to have a higher binding in rat striatum injected with a viral vector expressing human P2X7R than in control rats. This compound was also used in monkeys and showed a specific binding to P2X7R, as the concomitant use of the JNJ-42253432, a P2X7R antagonist, completely block the brain fixation of the radiolabeled compound [128]. Anyway, the use of ^11^C-JNJ-54173717 in a model of neuroinflammation has to be performed in order to validate its utilization as a marker of activated microglia. The other molecule recently tested is the GSK1482160, a strong P2X7R antagonist, evaluated in a phase 1 clinical study with a good BBB penetration [129]. The GSK1482160 has, thus, been labelled with carbon-11, and then evaluated in mice treated by LPS as a model of neuroinflammation, showing a significate increase of ^11^C-GSK1482160 binding in treated mouse brains vs. control [82]. Therefore, ^11^C-GSK1482160 appears as a promising radioligand of P2X7R, as a marker of neuroinflammation.

#### 5.1.4. β-Glucuronidase

β-Glucuronidase is a lysosomal enzyme involved both in the hydrolysis of glycosaminoglycans on the cell surface and in the degradation of the extracellular matrix. Several studies have reported an increase of β-glucuronidase expression by activated microglia into the extracellular space at the site of neuroinflammation [130,131]. Elevated levels of β-glucuronidase have been reported in the temporal cortex of AD patients and in the putamen of HD patients [132] and, thus, constituting a biomarker of neuroinflammation in relation with neurodegenerative diseases. Antunes et al. [83] have designed a PET tracer for β-glucuronidase imaging, namely ^18^F-FEAnGA, that, despite a moderate brain uptake, succeeded in detecting an increased release of β-glucuronidase during neuroinflammation in an encephalitis rat model.

#### 5.1.5. Adenosine Receptor 2A

Adenosine receptors belong to the purinergic G-coupled family receptors and are involved in inflammatory processes. Microglia express several types of adenosine receptors (A1, A2A, A2B, A3). Among them, the 2A adenosine receptor (A2AR) seems to have an important implication in neurodegeneration [133], is overexpressed in vitro in activated microglia, and is also involved in the regulation of microglia activation [134,135]. A2AR has been considered an interesting target of activated microglia.

Several tracers of A2AR have been synthetized (^11^C-TMSX, ^11^C-preladenant, ^18^F-FESCH) and clinical studies have been performed with the ^11^C-TMSX [84,136,137]. ^11^C-TMSX showed that A2AR is increased in MS patients, in association with neuroinflammation, with a binding correlated with the severity of symptoms and the loss of cerebral tissue[85]. However, in PD patients, the uptake of this tracer is not related to neuroinflammation, but to neuronal regulation [84]. A2AR are expressed both in neurons and astrocytes but, regarding their brain distribution, they are strongly expressed in the striatum (post-synaptic neurons) [84,138,139]. This striatal neuronal expression probably explains results obtained in PD patients [84]. However, A2AR’s increase in other brain areas may be relevant to follow brain inflammation elsewhere than in the striatum and might be therefore an interesting tool in neurodegenerative diseases such as MS.

#### 5.1.6. Nicotinic Acetylcholine Receptors α4β2

Nicotinic acetylcholine receptors (nAChR) are pentameric ligand gated ion channels. In the brain, several subtypes of nAChR have been identified and, among them, the heteromeric α4β2 is one of the most abundant. α4β2 receptor expression in microglia is not well characterized yet. The 2-^18^F-fluoro-A85380 compound has been synthetized in order to follow α4β2’s expression in neurodegenerative diseases [140]. Ex vivo studies with this tracer showed a decrease of binding in brain sections from AD patients [141], making this radiotracer a tool to follow the death of cholinergic neurons in neurodegenerative diseases. More recently, Martín et al. [86] used 2-^18^F-fluoro-A85380 as a marker of neuroinflammation in a model of cerebral ischemia. Their results showed an uptake of this tracer similar to the one of PK11195 (for TSPO PET imaging), confirming the inflammatory expression of α4β2 in this model. Moreover, authors confirmed by immunohistochemistry the overexpression of α4β2 in microglia and astrocytes. Nonetheless, this overexpression of α4β2 in activated microglia is not well characterized yet and the neuronal expression of α4β2 is a limiting factor for the use of the nAChR tracer to follow microglia activation in vivo.

#### 5.1.7. Matrix Metalloproteinases

Matrix metalloproteinases (MMPs) are a family of endopeptidases able to degrade the components of the extracellular matrix (collagen, gelatin, elastin) and are involved in tissue remodeling and degradation. Several subtypes of MMPs have been identified and their expression is enhanced by pro-inflammatory signals, such as cytokines (IL-1β, TNFα) or LPS. MMPs are associated in the CNS to excitotoxicity, neuronal damage, and BBB disruption, but also to the progression of neurodegenerative diseases [142,143]. Several tracers of MMPs or specific to one MMPs subtype have been synthetized in the last years.

Among them, the ^18^F-BR-351, derived from a non peptidic MMP inhibitor, was used in a rat model of stroke by transient middle cerebral artery occlusion (tMCAo) [88,144]. In this study, PET imaging of MMPs and TSPO were performed with the ^18^F-BR-351 and the ^18^F-DPA714, respectively. Authors showed time course expression of MMPs and TSPO following tMCAo by PET imaging and confirmed the expression of MMP-9 in microglia in the infarct brain part by immunofluorescence. The same compound has very recently been used in a mice model of gliomas [145]. In parallel, other MMPs inhibitors, specific of one or several MMPs, have been labeled with ^18^F or 11C and showed brain uptake in small rodent biodistribution. These tracers have not been tested in a pathological model yet [146,147]. 

### 5.2. Molecular Targets Evaluated in Other Inflammatory Diseases

Even if not tested for microglia imaging yet, the molecular targets cited here might be promising for PET microglia imaging (Table 2). For these targets, PET ligands have been developed and tested in peripheral inflammatory conditions (i.e., macrophages expression). Some of these targets could be specific to the M1 or M2 phenotypes, but this specificity has never been demonstrated by in vivo imaging. Furthermore, the capacity of their ligands to cross the BBB must be studied before any application related to microglia activation.

#### 5.2.1. Inducible Nitric Oxide Synthase

Nitric oxide (NO) is a ubiquitous cellular messenger, involved in several physiological processes in peripheral systems, but also in the brain. NO production by immune cells (macrophages, microglia) participates in innate responses, and especially in cell death, through the inhibition of mitochondrial respiration [156,157]. The inducible nitric oxide synthase (iNOS) that produces NO is not expressed in the brain, or at very low concentration, in physiological conditions but, in inflammatory conditions, iNOS is overexpressed in microglia and astrocytes [158]. iNOS is also considered as specific of a M1 phenotype in both macrophages and microglia [148,159]. iNOS macrophage’s expression in inflammatory condition has been assessed in vivo using the ^18^F6-(2-fluoropropyl)-4-methyl-pyridin-2-amine, both in mouse and human. It was tested in vivo in a mouse model of iNOS induction by LPS injection. Results showed a significantly higher uptake of the tracer in the lung of LPS mice vs. control [160]. More recently, the same compound, renamed ^18^F-NOS, was used in healthy volunteers for the first in-human evaluation. A comparison was made on subjects after endotoxin administration in the right lung, which evidenced an increase of 30% of ^18^F-NOS intake in this lung vs. the left one [149].

#### 5.2.2. Folate Receptor β

Folate receptor (FR) is a family of four receptor subtypes (α, β, γ or δ) that bind folic acid. FRβ expression is described in activated macrophages in the model of inflammatory diseases (rheumatoid arthritis, Crohn’s disease, etc.) [161]. FRβ is not expressed in quiescent or resting microglia. As FRβ award macrophages to internalize molecules derived from folic acid, the development of tracers derived from folic acid could allowed the following of activated macrophages and microglia [162]. Moreover, in vitro studies showed that FRβ is specifically expressed by M2 polarized macrophages [163]. In an in vivo rat model of restraint stress MacDowell et al. [164] used FRβ as a specific marker of M2 microglia. Nonetheless, this seems to be the only publication describing FRβ as a specific marker of M2 microglia. On the other hand, a study on macrophages isolated from mice after bacterial infection showed a correlation between FRβ expression, ROS production, and TNFα secretion, both of which were more specific to an M1 phenotype [165]. FRβ might be involved in microglia M1/M2 polarization, but extended studies on this receptor expression in microglial cells are still required to assess its interest for PET microglia imaging.

Kularatne et al. [150] developed two conjugated molecules derived from folic acid for FRβ PET imaging: 4-^18^F-fluorophenylfolate and ^68^Ga-DOTA-folate. They tested these compounds to follow macrophages activation in a model of paw inflammation in the rat and showed that uptake of both radiotracers was increased in the inflamed paw. Another compound, ^18^F-fluoro-PEG-folate, also gave interesting results in a rat model of rheumatoid arthritis [151]. If no clinical studies using PET tracers of FRβ have been performed yet, a single-photon emission computed tomography (SPECT) tracer, ^9m^Tc-EC20, was used on patients with rheumatoid arthritis [166].

### 5.3. Potential Targets of Microglia Activation

In this part, we regroup targets expressed in activated microglia but for which no radiotracer has been synthetized or tested yet (Table 2). More investigations are needed to assess in vivo expression of these targets in activated microglia.

#### 5.3.1. Enzymes of the Kynurenine Pathway: Indoleamine 2,3-dioxygenase-1 and Kynurenine-3-monooxygenase

The kynurenine pathway (KP) mediates the tryptophan catalyzation in both periphery and CNS. KP is stimulated by inflammatory molecules, such as IFN-γ, and, in the brain, products of KP have been identified to be neuroprotective (picolinic acid, kynurenic acid) or neurotoxic (quinolinic acid, 3-hydroxykinurenine) [167]. Indoleamine 2,3-dioxygenase-1 (IDO-1) is one of the limiting enzymes of KP, is expressed in immune peripheral cells and, in the brain, in microglia cells, astrocytes, and neurons. IDO-1 is strongly induced in primary microglial cells by LPS and IFN-γ [168] and is overexpressed during neurodegenerative disease processes, such as for MS [169], AD [170], and PD [171]. Furthermore, in AD patient brains, IDO-1 immunoreactivity is increased in microglia and astrocytes [172], making IDO-1 a serious candidate to follow microglia activation in vivo.

Several analogues of tryptophan have been synthetized, labelled, and used in vitro or in animal models with tumor grafts [173]. These molecules are promising to follow tryptophan metabolism but are not specific of IDO expression. Huang et al. [152] used another approach to develop a tracer of IDO-1 by using IDOL5, a strong antagonist of IDO-1. They successfully labeled IDOL5 with ^18^F but this potential tracer has not been evaluated neither in in vitro nor in vivo tests so far.

More recently, the kynurenine-3-monooxygenase (KMO), another enzyme involved in KP, has been described as regulated by proinflammatory cytokine signals. In vivo, in the brain, KMO is predominantly expressed in microglia [153] and is involved in the regulation of quinolinic acid production. KMO is overexpressed, in vitro, in macrophages and microglia activated by IFN-γ. KMO is also increased by LPS treatment in rat brains. KMO could be an interesting target to follow microglia activation, but its characterization requires more investigation.

#### 5.3.2. P2Y12 Receptor

The P2Y12 receptor (P2Y12R), a purinergic G-protein-coupled receptor, is exclusively expressed in microglia in the CNS. This receptor is not found in peripheral macrophages and is, thus, a good marker to distinguish resident microglia from infiltrated macrophages [174]. Expression of P2Y12R in activated microglia is still hard to qualify but showed that in vitro M2-polarized microglia overexpressed P2Y12R [155]. On the other hand, pathological conditions, such as AD, are associated with a decrease of P2Y12R expression in microglia, notably near the plaques or lesion sites, compared to microglial cells in other brain areas [174,175]. This decrease of P2Y12R expression in such inflammatory conditions (i.e., the presence of proteins able to activate the toll like receptor 4 (TLR4) pathway) may be associated with an increase of M1, and a diminution of M2, microglial cells in these areas. This hypothesis is supported by the P2Y12R expression in microglia of brains with parasite infections, a condition known to privilege M2 polarization of immune cells [155]. P2Y12R may, thus, be a promising target of M2 microglia but further studies are needed to confirm its in vivo expression in this microglia subpopulation.

## 6. Conclusions

It is, to date, broadly recognized that neuroinflammation, and in particular microglia activation, plays a crucial role in various brain disorders, from acute (stroke, TBI) to chronic (neurodegenerative disorders) ones. Initially, the large body of post mortem evidence of activated microglia in various CNS conditions led most of the authors to consider the microglial cells’ shift from sensing activity to a reactive state as a deleterious process. This microglial activation has been first assessed in vivo thanks to PET radioligands targeting TSPO. Polymorphism and multicellular expression of TSPO, as well as the lack of a specific brain region of negative control, led to identifying other molecular biomarkers of activated microglia that would likely be complementary to TSPO PET imaging. Among the targets of interest summarized in the present review, P2X7 receptor appears to be the most promising one regarding: (1) its implication in neurodegenerative diseases’ pathophysiology, highlighted by numerous current clinical trials aiming at evaluating P2X7 antagonists in CNS indications [176]; (2) the recent development of efficient dedicated radiopharmaceuticals that are currently coming available for clinical trials; (3) its implication on M1 microglial polarization. Thus, a multi-targets approach for PET imaging of microglia activation, combining TSPO radiopharmaceuticals with new probes specific of P2X7 expression may help to characterize the involvement of neuroinflammation over the CNS disorder’s progression, as well as to follow the effects of clinical treatments on microglia polarization. 

## Figures and Tables

**Table 1:** Activated microglia molecular targets with current applications for central nervous systems (CNS) disorders’ positron emission tomography (PET) exploration.

Target	Cellular Localization	Cellular Expression	Functions	M1/M2 Expression	Applications	References
**COX**	Cytoplasmic enzyme	Microglia, neurons	Prostaglandins synthesis	No data on microglia subtypes expression	COX-1 PET tracer: pre-clinical study on animal model of AD COX-2 PET in rat models of neuroinflammation	[77,78]
**CB2R**	G-protein-coupled receptor	Microglia, astrocytes, microvascular endothelial cells	Inhibition of pro-inflammatory cytokines’ (IL-1, TNF-α) release Activation of anti-inflammatory cytokines’ (IL-4, IL-10) release	No data on microglia subtypes expression	^11^C-NE40: in human study in AD vs. control patients (no increase of ligand binding in AD patients) Other ligands: preclinical studies such as brain uptake in healthy rodent or post-mortem binding in human ALS brain	[79,80]
**P2X7R**	Cation-permeable ion channel receptor	Microglia, macrophages, astrocytes, Schwann cells	Activation of pro-inflammatory cytokines’ (IL-1β) and ROS release	Potentially specific of M1 subtypes	In vivo preclinical study on LPS-induced neuroinflammation (rat)	[81,82]
**β-glucuronidase**	Lysosomal enzyme	Microglia, astrocytes, neurons	Anti-inflammatory effects	No data on microglia subtypes expression	In vivo preclinical study on an encephalitis rat model	[83]
**A2AR**	G-protein-coupled receptor	Microglia, astrocytes, neurons	Anti-inflammatory effects	No data on microglia subtypes expression	In human study on PD and MS	[84,85]
**α4β2 nAChR**	Pentameric nicotinic receptor	Microglia, neurons	Anti-inflammatory effects (cholinergic anti-inflammatory pathway)	No data on microglia subtypes expression	Preclinical study on neuroinflammation induced by cerebral ischemia (rat)	[86]
**MMPs**	Immature enzymes are cytoplasmic and secreted and activated extracellularly	Microglia, neurons, astrocytes, oligodendrocytes	CNS development including neurogenesis, myelogenesis, and axonal guidance.	No data on microglia subtypes expression	In vivo preclinical studies on a rat model of stroke	[87,88,89]

A2AR: adenosine receptor 2A; CB2R: cannabinoid receptor type 2; COX: cyclooxygenase; nAChR: nicotinic acetylcholine receptor; P2X7R: purinergic receptor 2 ion channel receptor; MMP: matrix metalloproteinases; IL: interleukin; TNF-α: tumor necrosis factor-α; ROS: reactive oxygen species; LPS: lipopolysaccharide; M1: Classically or pro-inflammatory activated microglia; M2: alternative or anti-inflammatory microglial cells; AD: Alzheimer’s disease; PD: Parkinson’s disease; MS: multiple sclerosis; ALS: amyotrophic lateral sclerosis

**Table 2:** Proposed alternative microglia molecular targets

Target	Cellular Localization	Cellular Expression	Functions	M1/M2 Expression	Applications	References
**iNOS**	Cytoplasmic enzyme	Microglia, macrophages, astrocytes	Immune innate response: NO production by immune cells	Potentially specific of M1 phenotype	In human study in healthy volunteers with endotoxin administration in one lung	[148,149]
**FRβ**	Surface receptor	Microglia	Captation and internalization of folic acid	Potentially specific of M2 phenotype	In vivo preclinical study on models of peripheral inflammation (paw inflammation, rheumatoid arthritis)	[150,151]
**IDO-1**	Cytoplasmic enzyme	Microglia, neurons	Tryptophan catalization	No data on microglia subtypes expression	Compound labelled but not evaluated in preclinical study	[152]
**KMO**	Cytoplasmic enzyme	Microglia, macrophages	Tryptophan catalization	No data on microglia subtypes expression	No PET tracer developed yet	[153]
**P2Y12R**	Purinergic G-protein-coupled receptor	Microglia	Involved in platelet agregation	Potentially specific of M2 phenotype	No PET tracer developed yet	[154,155]

COX: cyclooxygenase; FRβ: folate receptor β; IDO-1: indoleamine 2,3-dioxygenase 1; iNOS: inducible nitric oxide synthase; KMO: kynurenine-3-monooxygenase; MMPs: matrix metalloproteinases; P2Y12: purinergic ion channel Y12.
